# Activation of *Drosophila* hemocyte motility by the ecdysone hormone

**DOI:** 10.1242/bio.20136619

**Published:** 2013-11-14

**Authors:** Christopher J. Sampson, Unum Amin, Juan-Pablo Couso

**Affiliations:** JMS Building, University of Sussex, Brighton BN1 9QG, East Sussex, UK

**Keywords:** Cell culture, Cell migration, Cytoskeleton dynamics, *Drosophila*, Ecdysone, Hemocytes

## Abstract

*Drosophila* hemocytes compose the cellular arm of the fly's innate immune system. Plasmatocytes, putative homologues to mammalian macrophages, represent ∼95% of the migratory hemocyte population in circulation and are responsible for the phagocytosis of bacteria and apoptotic tissues that arise during metamorphosis. It is not known as to how hemocytes become activated from a sessile state in response to such infectious and developmental cues, although the hormone ecdysone has been suggested as the signal that shifts hemocyte behaviour from quiescent to migratory at metamorphosis. Here, we corroborate this hypothesis by showing the activation of hemocyte motility by ecdysone. We induce motile behaviour in larval hemocytes by culturing them with 20-hydroxyecdysone *ex vivo*. Moreover, we also determine that motile cell behaviour requires the ecdysone receptor complex and leads to asymmetrical redistribution of both actin and tubulin cytoskeleton.

## Introduction

*Drosophila melanogaster* is a widely studied model organism in the context of genetics and development, and also presents great advantages for cell biology research. The whole organism develops over a 10 day life cycle starting from a 24 hour embryogenesis, advancing through three larval instar stages then undergoing a second stage of morphogenesis at the pupal stage before eclosing as an adult. Coinciding with these developmental transitions of *D. melanogaster* are peaks and troughs of hormone expression that precede the onset of various morphogenetic changes (Cherbas et al., 1989; Thummel, 1996). The most expansive stage of morphogenesis is the pupal metamorphosis, during which the whole organism undergoes massive changes in tissue morphology and development through cell proliferation and apoptosis, which form the winged adult from a crawling larval maggot. These processes require a cellular workforce and also a central signalling, and organising, network to orchestrate the behaviour of multiple cell types at the onset of, and during, these stages of morphogenesis.

Ecdysteroid 20-hydroxyecdysone (ecdysone) is a moulting hormone that regularly surges between each moulting life stage, with the largest peak occurring at puparium formation (supplementary material Fig. S1). Ecdysone signalling is mediated through interaction with the nuclear ecdysone receptor (EcR). The gene *ecr*, encodes three receptor isoforms, EcR-A, EcR-B1 and EcR-B2, that form heterodimers with the *Drosophila* retinoid X receptor (RXR) homologue Ultraspiracle (USP) (Yao et al., 1993). The ecdysone-EcR/USP complex interacts with ecdysone responsive elements (EcREs), DNA enhancer sequences which control temporal and spatial gene expression of the target genes (Ashburner, 1974; Hill et al., 2013). This regulation of transcription occurs across all tissue and cell types at the larval to pre-pupal transition and triggers widespread apoptosis of larval tissues and proliferation of various cell types that are necessary for the formation and emergence of the adult tissues (Rusten et al., 2004).

*Drosophila* hemocytes are motile, phagocytic cells that are present at all stages of the life cycle and represent the cellular component of the animal's innate immune system at post-embryonic life stages (Lanot et al., 2001). The most common cell type is the plasmatocyte, which exhibits cytoskeletal behaviours mediated by conserved pathways involving the Rho family GTPases, similar to those of *Drosophila* embryonic hemocytes, neutrophils and mammalian macrophages (Cox and Huttenlocher, 1998; Meister, 2004; Wood et al., 2006; Bretscher, 2008; Williams, 2007). Accordingly, the main function of post-embryonic hemocytes is to clear infection from invading microorganisms as well as debris from apoptotic cells by performing phagocytosis (Holz et al., 2003; Lanot et al., 2001). These cells extend protrusions of actin-rich filopodia and lamellipodia to migrate on various extracellular matrices to reach their destinations, guided by extracellular cues that are still to be fully identified (reviewed by Wood and Jacinto, 2007).

There are two haematopoietic waves during *Drosophila* development – one occurring during embryogenesis and the other from the transient lymph gland at puparium formation (Tepass et al., 1994; Jung et al., 2005). This correlates with distinct developmental roles for hemocytes at relevant developmental time points, indicating that these cells are responsive to triggers of developmental timing (reviewed by Wood and Jacinto, 2007). Broadly speaking, in the overall course of development, a motile embryonic population changes into a largely sessile and inactive population at larval stages (forming a reservoir for release in case of infection), and then back to a highly motile and phagocytic state at puparium formation (Lanot et al., 2001; Holz et al., 2003; Crozatier and Meister, 2007; Honti et al., 2009; Márkus et al., 2009; Wood et al., 2006) (supplementary material Fig. S1).

By using a novel *ex vivo* primary cell culture technique, a study of morphological classes of hemocytes extracted at different developmental stages revealed the expected ‘switch’ of hemocyte morphology and behaviour between larval (LIII) and white pre-pupal (WPP) stages. LIII hemocytes are sessile and quiescent but they are activated towards an exploratory behaviour at WPP, similar to the behaviour of embryonic hemocytes (Evans et al., 2010; Sampson and Williams, 2012a; Sampson and Williams, 2012b). This activation does not take place in mutants affecting cytoskeletal reorganisation such as Cdc42, Rac1 and Rac2 (Sampson and Williams, 2012b).

It has been proposed that the change in cell behaviour at the LIII to WPP transition could be due to the surge of ecdysone at the onset of metamorphosis (Lanot et al., 2001; Sampson and Williams, 2012b). This hypothesis remains unproven and, further, questions arise as to whether the induction of cell motility would be due to the direct effect of ecdysone on the hemocytes or an indirect effect caused by ecdysone-dependent secondary signals from other tissues.

To answer these questions, we have cultured primary *Drosophila* post-embryonic hemocytes with ecdysone to recapitulate the developmentally induced change in cell activity at the onset of metamorphosis. First, we have analysed morphology, migration capability and cytoskeletal polarisation of the hemocytes at larval and pre-pupal stages. Second, we determine the effect of adding ecdysone to LIII hemocytes and show that there is a shift from rounded morphology and immotile behaviour to polarised, motile cell population similar to WPP hemocytes. Reciprocally, we have also checked the effect of EcR dominant negative forms, and we have found that they block this hemocyte activity.

## Results

### The ecdysone hormone changes hemocyte morphology

**Fig. 1.**
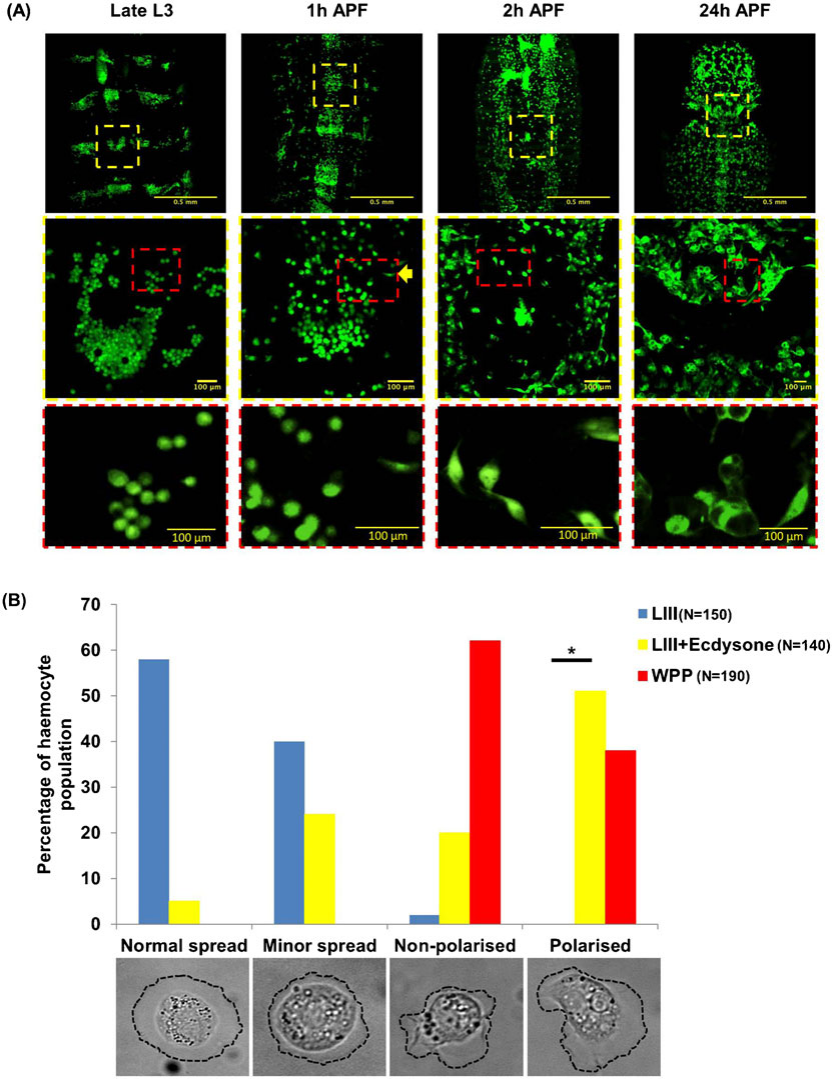
Ecdysone causes a shift in hemocyte morphology and distribution. (A) Images of the presumptive thorax/abdominal region with hemocytes labelled by PxnGAL4-UASGFP; CrqGAL4-UASGFP. At the Late L3 stage, hemocytes are attached to the larval integument and are localized to segmental patches and have rounded morphology (yellow dashed squares in the low-magnification top rows indicate the region magnified in the middle panels (2× digital zoom); in these medium-magnification middle row panels, the red dashed squares indicate the region magnified (2× digital zoom from middle panels) in the high-magnification panels in the bottom row). At 1 h APF, the hemocytes have started to migrate out of these patches and begin to assume polarized morphology (middle panel, yellow arrow head). The zoomed image presented in the yellow box, under 1 h APF, was taken from the last frame of a live time-lapse and at the most ventral position within the Z-stack. This image was chosen as the best representative of hemocyte patch dispersal. At 2 h APF, hemocytes have migrated laterally from the dorsal patches as well as detached from the integument, as they are no longer in view at the dorsal plane of focus. Most of the population has adopted a spindle/polarized shape (bottom panel). At 24 h APF the hemocytes have colonized most of the thorax and abdomen and display vacuolation associated with phagocytosis of larval tissues and secretion of ECM. These *in vivo* morphologies are akin to those found in *ex vivo* culture (see B). (B) A histogram showing the percentages of cellular morphologies and behaviours between LIII (−ecdysone) (blue bars), WPP (red bars), and LIII +ecdysone (yellow bars) hemocyte populations *ex vivo*. Black bar with a star (*) represents significant difference. Sample images of each morphological class are shown at the bottom.

One of the most striking changes we observed *in vivo* at puparium formation was the rapid colonization of the entire body by hemocytes that were previously sessile and attached to the larval integument (Fig. 1A). At late LIII stage there were very few hemocytes circulating in the body (supplementary material Movie 1) but a few minutes after spiracle eversion, a primary sign of pupation, the hemocytes were observed to increase in number and redistribute from the dorsal patches, migrating towards target tissues (Fig. 1A; supplementary material Movie 4). Simultaneously, hemocytes changed from rounded to spindle shaped cells as the animal transitions between late LIII and early WPP. These observations could not fully distinguish if the hemocytes were moved by self-propulsion or by tissue rearrangements and hemolymph currents, but altogether show marked changes in the state of hemocyte population at the onset of metamorphosis.

In order to ascertain and quantify the true cellular effects of this transition, and to explore the role of ecdysone, we exploited the *ex vivo* primary culture system, which allows us to observe (1) classes of hemocytes by their cellular morphologies, (2) self-polarised migration on synthetic ECM, and (3) cytoskeleton dynamic behaviour. Four classes of hemocyte morphology were used from Sampson and Williams (Sampson and Williams, 2012b). As a brief description, the most common morphology at LIII stages ‘normal spread’ represented a fully adhered cell with a uniformly protruding and retracting cell membrane (at all angles surrounding the centroid region). The next common LIII stage morphological class was ‘minor spread’, which represented a fully adhered cell with a uniformly protruding and retracting cell membrane (similar to normal spread) but the membrane edge is visually in close proximity to the cell centroid region. Both normal and minor spread represented the quiescent sessile behaviour of these hemocytes. The two active morphological classes, ‘non-polarised’ and ‘polarised’, are predominant at WPP stages. Both classes represent a fully adhered cell that exhibits an extremely active cell membrane that protrudes and retracts at varied angles to the cell centroid region producing multiple membrane ruffles. The essential difference between these two classes is that polarised cells produce a visual lamellipodium and trailing edge during random migration (Fig. 1B).

*Ex vivo* larval hemocytes (Fig. 1B, blue bars; supplementary material Movie 2) exhibited 40% minor plasma membrane spread, ∼58% normal spread, ∼2% non-polarised spread and membrane ruffles, and 0% polarised ruffles and spread forming a lamellipodium, (and a trailing edge at the opposite cell pole) (Fig. 1B). In contrast, WPP hemocytes (Fig. 1B; supplementary material Movie 3) exhibited only non-polarised and polarised morphologies at ∼62% and ∼38%, respectively. Upon addition of ecdysone to the culture, LIII hemocytes showed a shift in hemocyte morphology and behaviour similar to that observed in WPP hemocytes (Fig. 1B, red bars), LIII +ecdysone hemocytes became non-polarised and polarised at ∼19% and ∼50%, respectively (Fig. 1B). Although an observable shift in morphology and behaviour was observed (supplementary material Movies 2 and 5), there was still a small number of hemocytes of normal and minor spread morphologies at ∼7% and ∼24%, respectively. Using a 2-sample poisson rate test, there was a significant difference (*P*<0.05) determined between LIII (*n* = 150 (total number of hemocytes in sample population)) and LIII +ecdysone (*n* = 140) hemocytes for the number of hemocytes presenting a polarised morphology with lamellipodium and trailing edge. Moreover, there was no significant difference (*P*>0.05) in polarised morphology found when LIII +ecdysone and WPP hemocytes were compared.

### Ecdysone induces polarization and migratory behaviour in LIII hemocytes

**Fig. 2.**
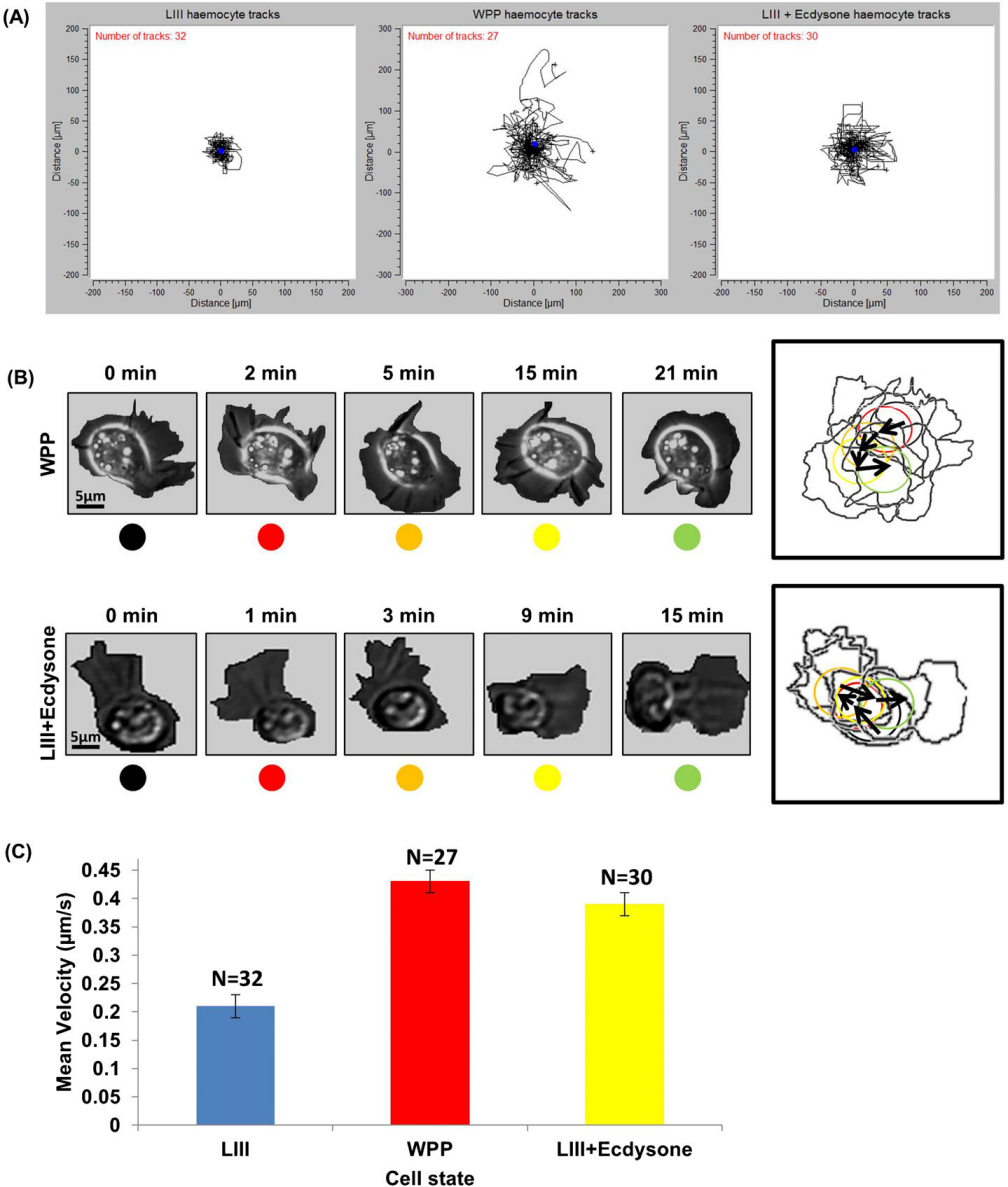
*Ex vivo* ecdysone hormone induces motile membrane behaviour in early LIII hemocytes. (A) A series of fixed point track plots from LIII, WPP, and LIII +ecdysone hemocyte populations in *ex vivo* cultures. Both X and Y axis represent distance in µm. (B) A collage of sample WPP and LIII +ecdysone hemocytes *ex vivo*. The box to the right contains an outline of each still overlaid with directional arrows; circles, that are colour coded corresponding to each time point in the collage, indicates the approximate centroid region of the cell. (C) Histogram showing mean migration velocity and standard deviation exhibited across the hemocytes analysed.

Next, we compared migratory behaviour between LIII and WPP stages. *Ex vivo* WPP hemocytes exhibit random cell migration in correlation with a highly active plasma-membrane capable of protrusion and retraction at a high rate, in multiple vectors around the cell (Sampson and Williams, 2012b). Such random migration is highly confined to a fixed spatial region surrounding the point of adhesion and does not show the long-range directed migration visualised *in vivo*, but it still displays formation of a biased lamellipodium in the direction of cell motion and moderate displacement of the cell body (supplementary material Movie 3). WPP hemocytes executed multiple turns, and at each turning point the cell stopped moving and re-polarised towards the new direction of travel before resuming movement (Fig. 2A,B; supplementary material Movie 3). LIII hemocytes that had been ecdysone treated exhibited similar behaviours to WPP (Fig. 2A,B; supplementary material Movie 5), such as polarisation and turning behaviour, that was not observed at the LIII.

Track plot analysis revealed further similarities between WPP and LIII +ecdysone during random migration. Fig. 2A shows fixed point track plots of individually tracked cells from LIII and LIII +ecdysone. LIII hemocytes showed minor displacement of 40 to 60 µm (Fig. 2A, left; supplementary material Movie 2). LIII +ecdysone hemocytes exhibited a dispersed exploratory behaviour similar to that of WPP cells (Fig. 2A, centre and right; supplementary material Movies 3 and 5) and these hemocytes presented an increased displacement ranging from 60 to 160 µm (Fig. 2A, right track plot). An analysis of migration velocity showed that LIII +ecdysone hemocytes migrated at velocities ranging from 0.14–0.81 µm/s, with a mean migration velocity of 0.39 (SE±0.02) µm/s. This was comparable to WPP hemocyte mean migration velocity at 0.43 (±0.02) µm/s, and also closely similar to previously reported WPP *in vivo* migration velocities (Moreira et al., 2013). There was significant difference (*P*<0.001), determined by a Student's T-test, to LIII hemocyte mean migration velocity at 0.21(±0.01) µm/s (Fig. 2C). Thus addition of ecdysone in the *ex vivo* system increases hemocyte migration.

In summary, this set of live data shows an increase of hemocyte activity and migratory behaviour at WPP stage of *Drosophila* development, which coincides with the developmental pulse of ecdysone at the onset of metamorphosis. Moreover, *ex vivo* cell culture of LIII hemocytes with ecdysone generates similar migratory behaviour to WPP hemocytes, indicating that the ecdysone hormone induces hemocyte activation.

### Ecdysone induces cytoskeletal re-arrangements in LIII hemocytes

**Fig. 3.**
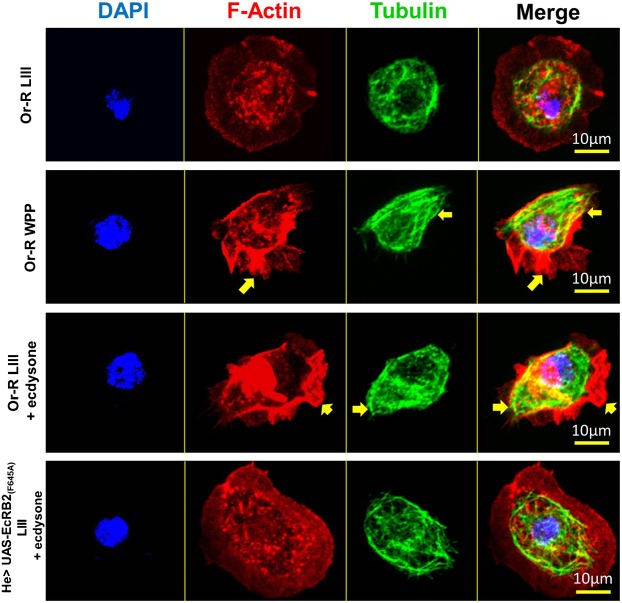
Cytoskeletal analysis showing actin and microtubule distribution in LIII, WPP, LIII +ecdysone and LIII EcRB2_(F645A)_ +ecdysone hemocytes. Fixed hemocyte images, taken at 63× magnification, showing DAPI, F-actin, and β-tubulin staining. DAPI (blue) labels the nucleus of the cell, phalloidin (red) labels polymerised actin (F-actin), and β-tubulin (green) labels the microtubules of the cell; merged images on the right. Representative hemocytes were selected from Or-R LIII, Or-R WPP, Or-R LIII + ecdysone, and *He_GAL4* UAS-EcRB2_(F645A)_ LIII + ecdysone. Arrows point out the presence of asymmetrical actin and microtubule cytoskeletal sub-units in polarised hemocytes. Scale bar is to 10 µm.

The induction of polarised cell behaviour and random migration is only possible by the breakdown of internal cytoskeletal symmetry followed by re-distribution of dynamic cytoskeletal components, toward opposite poles of the cell, such as the leading edge and the trailing edge (Bretscher, 2008). Ecdysone induces such cytoskeletal changes in the plasma-membrane region of both WPP and LIII +ecdysone hemocytes (Fig. 2B; supplementary material Movies 3 and 5). Underlying this membrane behaviour we observe that WPP and LIII +ecdysone hemocytes exhibit asymmetrical distribution of both actin and tubulin in fixed cells (Fig. 3).

F-actin appeared asymmetrically distributed at the plasma-membrane in WPP and LIII +ecdysone hemocytes, forming an actin rich region that was representative of lamellipodium formation (Fig. 3, WPP and LIII +ecdysone, yellow arrows, F-actin only) (Stramer et al., 2010), and which was not observed in LIII hemocytes. LIII hemocytes did not produce F-actin rich regions at the plasma-membrane, but rather actin-rich spots located around the nucleus in the cytoplasmic region of the cell (Fig. 3, *Or-R* LIII, F-actin only).

**Fig. 4.**
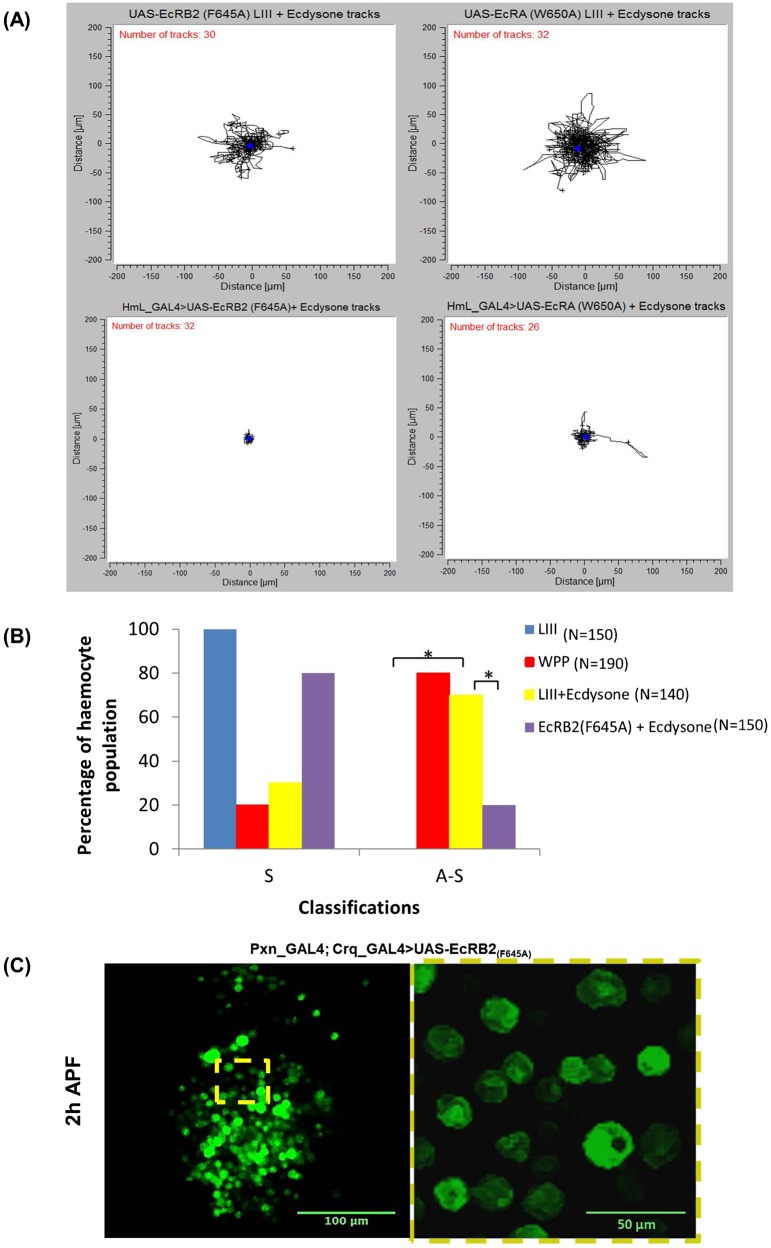
The ecdysone receptor is required for hemocyte motility. (A) Top left and right panels show a pooling of *UAS-EcR-dominant-negative* control tracks, whilst bottom left and right show the respective experimental results when these constructs, *UAS-EcRB2_(F645A)_* and *UAS-EcRA_(W650A)_*, are expressed by the hemocyte specific driver *HmL_GAL4* (see supplementary material Fig. S2 for the *He_GAL4* driven *UAS-EcRB2_(F645A)_* track plot). (B) Percentages of hemocytes that exhibited either a symmetrical or an asymmetrical cytoskeletal phenotype amongst the four groups tested previously shown in Fig. 3 – Or-R LIII (blue bars), Or-R WPP (Red bars), Or-R LIII +ecdysone (yellow bars) and *He_GAL4* driven *UAS-EcRB2_(F645A)_* LIII +ecdysone (Purple bars). ‘N’ indicates the number of hemocytes tested in each sample population, ‘S’ indicates symmetry, ‘A–S’ asymmetry and the (*) indicates significant differences between the bracket bars. (C) *UAS-EcRB2_(F645A)_* expressed *in vivo* in hemocytes precludes the changes in morphology and distribution observed in wild-type hemocytes at WPP (compare with second and third rows of Fig. 1A).

Tubulin was also asymmetrically distributed along the length of the cell (Fig. 3, WPP and LIII +ecdysone, yellow arrows, tubulin only). This microtubule array was characteristic of cytoskeletal behaviour after the breaking of symmetry during cell polarisation. In LIII +ecdysone hemocytes, the tubulin cytoskeleton was also elongated from the cytoplasmic region through to the plasma-membrane edge, unlike in LIII hemocytes that exhibited a symmetrical tubulin cytoskeleton that was normally limited to the cytoplasmic region (Fig. 3, *Or-R* LIII, tubulin only). The number of hemocytes that showed symmetry and asymmetry were quantified and significant difference (*P*<0.05), using a 2-sample poisson rate test, was determined between *Or-R* LIII +ecdysone (*n* = 140) against LIII (*n* = 150) (Fig. 4B).

To summarize, we observed that LIII +ecdysone and WPP hemocytes displayed similar cytoskeletal distribution of F-actin and tubulin *ex vivo*, which also correlated with comparable migration velocities, as well as morphological classes (Fig. 1B) between both groups. It appears that addition of ecdysone to LIII hemocytes can induce similar changes in cellular behaviour and architecture to those displayed by WPP hemocytes after their own exposure to ecdysone *in vivo* at the onset of metamorphosis. These results also show that activation of hemocytes by ecdysone does not require a secondary signal from surrounding tissues, which are absent in the *ex vivo* system.

### Dominant-negative EcR blocks ecdysone-induced hemocyte motility and cytoskeleton dynamics

The ecdysone receptor is a heterodimer complex composed of the EcR protein and the ultraspiracle protein (USP). The EcR protein can be present in three isofroms -A, -B1, and -B2, that differ only at the N-terminus A/B domain, which is highly variable in amino acid length and sequence (Cherbas et al., 2003). Upon binding of ecdysone to the ecdysone receptor, the complex binds DNA and controls the transcription of the target genes. In most tissues this activation is mediated by the C-terminal AF2 domain, that contains an ecdysone-binding domain and a DNA-binding domain, and that is highly conserved across all three isoforms (Hu et al., 2003). The ecdysone-response genes contain ecdysone-response elements (EcREs) in their regulatory regions, and their transcription can be either up- or down-regulated in this way (Dalton et al., 2009; Thummel, 1996).

In order to corroborate the direct effect of ecdysone hormone on hemocyte behaviour and motility, the GAL4-UAS system was used to express two different dominant negative forms of EcR: *UAS-EcRA_(W650A)_* and *UAS-EcRB2_(F645A)_* (Cherbas et al., 2003) using two different hemocyte-specific Gal4 drivers. These EcR dominant-negative forms behave as competitive inhibitors of all the endogenous wild-type EcR isoforms (EcR-A, -B1 and -B2) for binding to USP and EcREs (Cherbas et al., 2003; Brown et al., 2006), therefore our approach should reduce overall ecdysone activation within hemocytes, regardless of which receptor isoform they actually expressed endogenously. Flies carrying these UAS-constructs but no GAL4 drivers provided the control LIII +ecdysone hemocytes, which as before were fully capable of ecdysone-induced motility and exhibited random migration (Fig. 4A, top left and right; supplementary material Movie 6).

Upon HmL_GAL4-driven expression of the dominant negative EcR-B2_(F645A)_ there was a loss of ecdysone-induced motile behaviour in LIII +ecdysone hemocytes (Fig. 4A, bottom left; supplementary material Movie 7). The same results were obtained when the other hemocyte-specific Gal4 line, He_GAL4, was used (supplementary material Fig. S2A; Movie 9). Moreover, in these hemocytes, F-actin and tubulin cytoskeletal polymers were symmetrically distributed throughout the cell after ecdysone incubation, which revealed an absence of cytoskeletal, and cell, polarization (Fig. 3). There were no areas that were differentially enriched in F-actin or tubulin, no lamellipodium formation nor formation of microtubule arrays along the length of the cell in EcRB2_(F645A)_ LIII +ecdysone hemocytes, in either HmL_GAL4 or He_GAL4 driven lines (Fig. 3; supplementary material Fig. S2B). These experiments showed a significant decrease (*P*<0.05) in the number of EcRB2_(F645A)_ hemocytes (*n* = 150) that were asymmetric (polarized) when compared to *Or-R* LIII +ecdysone hemocytes (*n* = 140) (Fig. 4B). Finally, a reduction in hemocyte motility and loss of cytoskeletal polarization were also observed in HmL_GAL4 UAS-EcR-A_(W650A)_ dominant negative hemocytes, although some hemocytes were still capable of random migration (Fig. 4A; supplementary material Fig. S2B; Movie 10).

We were also able to recapitulate these results *in vivo* with the hemocyte specific Pxn_GAL4; Crq_GAL4 line that expressed these dominant negative forms of EcR. When observed after puparium formation (Fig. 4C; supplementary material Movie 8), the GFP labelled hemocytes of flies expressing dominant negative EcR-A_(W650A)_ and EcR-B2_(F645A)_ lacked the movement and morphological changes observed in wild-type hemocytes at the same age (Fig. 1). This result implied that the changes in hemocyte distribution and morphology observed in wild-type pupae (Fig. 1) arose from a cell-autonomous response of hemocytes to ecdysone, at least entailing an active detachment of hemocytes from their larval reservoirs and possibly involving self-propelled migration as well.

In conclusion, we observed an overall reduction in ecdysone-induced motile behaviour in hemocytes, whether we used either of the drivers He_GAL4, HmL_GAL4 or the Pxn_GAL4; Crq_GAL4 combo, whether we expressed either of two different dominant negative forms of the ecdysone receptor, and whether we observed hemocytes *in vivo* or *ex vivo*. The simplest interpretation is that DN-EcR blocks ecdysone-induced motility, and hence that activation of hemocytes follows ecdysone signalling mediated through the Ecdysone-EcR/USP signalling complex.

## Discussion

The hormone 20-hydroxyecdysone acts as a developmental signal during metamorphosis instructing fate changes in multiple cell types, thus leading to broad changes to the body plan of the whole organism. At metamorphosis, the hemocyte population increases in number and change from sessile in the larva to generally circulating freely in the haemolymph of adults (Lanot et al., 2001). It is also known that in response to injury and/or infection, embryonic hemocytes can become motile, migrate to injury sites and become actively phagocytic in response to specific signals released by necrotic cells such as inflammation factors induced by extracellular hydrogen peroxide and calcium (Ca^2+^) (Wood and Jacinto, 2007; Williams, 2007; Razzell et al., 2013). However, the molecular trigger for the developmental switches from quiescent larval to motile at the onset of metamorphosis is still unknown. Ecdysone was suggested as a possible trigger (Sampson and Williams, 2012b) but this possibility has remained untested till now.

During *Drosophila* metamorphosis, organs such as the fat body undergo apoptosis (Rusten et al., 2004; Abrams et al., 1993). Clearance of the apoptotic cells contributes to the refurbishment of the overall body plan and is one of the main functions of circulating plasmatocytes (Tepass et al., 1994; Lolo et al., 2012). It could be postulated that high levels of apoptosis release signals which could induce cell migratory behaviour in the hemocyte population as a whole. However, we show here that ecdysone induces larval hemocyte motility *ex vivo*, i.e. in the absence of apoptosis in surrounding tissues or in hemocytes themselves. Further, this result indicates that such a response does not involve any secondary signals produced by other tissues.

This action of ecdysone in hemocytes appears mediated through the EcR/USP receptor complex. Dominant negative versions of the EcR-A, EcR-B1 and EcR-B2 isoforms have been reported to cause local and global phenotypes that were overlapping in some instances and seemingly specific in others. In principle, different cell types across different cellular populations, or possibly even within the same population, may possess different ratios of EcR isoforms, which may be linked to those cells' response to ecdysone (Davis et al., 2005). EcR-B1 and EcR-B2 differ considerably in their N-terminal amino acid sequence and length but they are both able to rescue EcR-B1 and -B2 dominant-negative specific phenotypes in neuronal pruning, leading to the hypothesis that they are functionally redundant (Brown et al., 2006). A specific phenotype was the loss of border cell migration during transition of stage 8 to stage 9 of the egg chamber during oogenesis (Cherbas et al., 2003). Although the phenotype was induced by dominant negative versions of all the three EcR isoforms, it could only be rescued by co-expression of functional EcR-B2. This might coincide with our observation that EcR-B2 dominant negative affects more strongly hemocyte motility and cytoskeletal re-organization, but given the reported generic effect of the EcR-dominant negative forms we have used (Cherbas et al., 2003; Brown et al., 2006) this could also be due to higher level of expression of the UAS-EcRB2_(F645A)_ versus the UAS-EcR-A_(W650A)_ insertion.

Our results do not formally exclude that hemocyte activation includes a hemocyte–hemocyte autocrine signalling acting downstream of the EcR/USP complex. Nonetheless, an EcR-dependent autocrine signalling that automatically follows EcR activation can be considered part of the hemocyte response to ecdysone signalling, with ecdysone still being the ultimate causal agent for such response. Eventually, clarification of the mechanism of action of ecdysone will require identification of the genes directly targeted by the EcR/USP complex. In order to implement changes in cell motility, ecdysone signalling must eventually impinge on the cellular machinery controlling the cytoskeleton. This machinery is regulated by post-transcriptional mechanisms involving protein–protein interactions. However, ecdysone binding to the EcR-USP complex controls transcription through direct binding to specific Ecdysone-response elements in the regulatory regions of the target genes (Thummel, 1996). How can this transcriptional regulation lead to cytoskeleton regulation?

Our data show a change in the actin and microtubule networks in response to ecdysone signalling, and data in the literature allow us to establish a link between these factors (supplementary material Fig. S3). It has been recently shown that *ex vivo* hemocyte motility requires the function of the actin regulators Cdc42, Rac1 and Rac2 (Sampson and Williams, 2012b). The function of Rac1 and Rac2 was more strongly required, possibly in correlation with their requirement for lamellipodia formation, in these cells (Williams, 2007; Paladi and Tepass, 2004). Further, it was previously shown that ecdysone negatively affects the levels of a Rac1 GTPase activating protein (GAP) *(rhoGAP16F)* in *Drosophila* Kc167 cells (Gonsalves et al., 2011). Altogether these data may suggest that ecdysone signalling regulates Rho family GAP levels in order to alter the ratio of GTPases toward a GTP-bound active state and thus control cell-wide levels of Rac1 and Rac2 activity (supplementary material Fig. S3).

In addition, an independent regulatory mechanism is suggested by the finding that ecdysone up-regulates the PI3K/MTOR pathway (Rusten et al., 2004; Insall and Weiner, 2001). This up-regulation could lead to greater production of phosphor-inositol lipids such as phosphatidylinositol (3,4,5) – triphosphate (PiP_3_) and greater of levels of ATP synthesis (Fader et al., 2012) which could result in up-regulation of Rac-GTPases and a higher turnover of membrane lipid production (supplementary material Fig. S3). Interestingly, PI3K is required for migration of embryonic hemocytes to wound sites (Wood et al., 2006) and has been recently shown to be required for hemocyte accumulation and phagocytic activity at the larval proventriculus (Zaidman-Rémy et al., 2012). Finally, ecdysone has also been shown to positively affect expression of the gene *rhea* that codes for the protein Talin, an actin binding protein that promotes focal-adhesions by clustering of integrins and actin–integrin linkage, which affects the regulation of cell shape and migration (Beckstead et al., 2005; Moreira et al., 2013).

These possible molecular mechanisms implemented downstream of ecdysone signalling remain currently speculative. However, hemocytes provide a suitable cell population where to study them using distinct, well described, and measurable motility and migratory responses (Wood et al., 2006; Stramer et al., 2005). By providing *ex vivo* corroboration of *in vivo* observations, we can study the effects of Ecdysone, (or any other cell signal), in isolation from the confounding effects of putative secondary signals from other tissues. Reciprocally, the triggering of motility in cultured hemocytes by ecdysone provides another *Drosophila* experimental system to study the control of cell motility in general, and in particular, the activation of macrophages in health and disease, in parallel to wound healing and *in situ* developmental studies in *Drosophila*.

## Materials and Methods

### Fly strains

All fly strains were acquired from the Bloomington stock centre, kept at 25°C on a standard cornmeal diet. Initial ecdysone studies were applied to hemocytes isolated from the lab wild-type standard strain, O*regon red* (*Or-r*). Ectopic expression in post-embryonic hemocytes used previously described *Hemese_GAL4* (*he_GAL4)* (Kurucz et al., 2003) and *Hemolectin_GAL4* (*hml_GAL4*) (Goto et al., 2001) to express dominant negative ecdysone receptors *UAS-EcRB2_(F645A)_* and *UAS-EcRA_(W650A)_*, respectively, previously described by Brown et al. (Brown et al., 2006) and Cherbas et al. (Cherbas et al., 2003). For *in vivo* whole larval and pupal mounts, to visualise UAS-GFP hemocytes, the hemocyte specific drivers *peroxidasin_GAL4* (*pxn_GAL4*) and *croquemort_GAL4* (*crq_GAL4)* (Wood et al., 2006) were used. *He-Gal4 UAS-EcRA_(W650A)_* individuals died as early larvae before hemocytes could be collected.

### *Ex vivo* cell incubation technique, with ecdysone

The *ex vivo* cell incubation technique was previously described by Sampson and Williams (Sampson and Williams, 2012a; Sampson and Williams, 2012b). Ecdysone [Sigma – H5142 5MG] was re-dissolved in 100% EtOH, to a working solution of 7.5 µg/mL in sterile *Drosophila* hemocyte isolation media (DHIM). For our purposes we used 7.5 µL of ecdysone stock in 300 µL of DHIM to create eDHIM at a 10% (v/v). Bled hemocytes were incubated for ≥3.5 hrs at 25°C in eDHIM.

### Primary hemocyte immunocytochemistry

The staining protocol was adapted from Zettervall et al. (Zettervall et al., 2004), whereby hemocytes were stained directly from *ex vivo* conditions therefore having undergone contact, adhesion and spreading across a 2D extra-cellular matrix (ECM), in this case collagen IV. All steps in this staining procedure were done at a maximum volume of 200 µL. Mouse anti-β tubulin primary [DSHB] was used at 1:500 df. Anti-mouse Alexafluor-488 secondary [Invitrogen, A11001], was also used at 1:500 df.

### *Ex vivo* imaging and Image analysis

An inverted Zeiss Axiovert 200M with automated prior stage was used to capture live imaging data using a Hamamatsu ORCA-ER C4742-95. *Ex vivo* time lapses were ≥20 minutes at 15 seconds/frame, as conducted previously by Sampson and Williams (Sampson and Williams, 2012b). Freeware software ImageJ v1.46r, with the plugin ‘Manual tracking’ was used to analyse the time-lapse movies.

### Experimental design and statistics

All *ex vivo* experiments consisted of hemocytes excised and isolated from a minimum of 6 larva/WPP, which was repeated a minimum of three repeats per life stage/experimental group. From this design two assays were conducted, morphological classification and migration velocity. Morphological classification utilised a 2-sample poisson rate test, whilst hemocyte migration velocity utilised a Student's T-test. All statistical tests were conducted using IBM SPSS statistics 20 software.

For the morphological classification, the 2-sample poisson rate tested the frequency of polarised hemocytes or asymmetrical hemocytes across all test groups. This used a sample population that consisted of all counted hemocytes from across all three experimental repeats. An ‘N’ of 100 (*n*≥100) cells/test group was considered a statistically safe minimum.

For the migration velocity, the 2-sample Student's T-test used a different set-up of hemocyte sample population in comparison to the morphological classification. A sample population that consisted of a minimum of 25 (*n*≥25) randomly selected hemocytes, from across all three experimental repeats, was used to determine statistical significance between respective average migration velocities.

### *In vivo* imaging and Image analysis

Larvae, white pre-pupae and brown pupae were mounted ventral-side down on double sided tape applied to a glass slide. Brown pupae were dissected in a manner similar to that of Moreira et al. (Moreira et al., 2011), by removing a window in the pupal case over the thorax. Larvae and WPP were imaged directly through the cuticle. After application of 10S Voltalef Oil on the region of interest, a ring of petroleum jelly was made around the samples, a coverslip was rested on this ring, and pressed down on the sample. The slide was raised on the microscope stage by adding additional pieces of glass slide to the edges of the slides to allow deeper focal length. Imaging was conducted on an inverted Zeiss LSM 510 with automated prior stage, using a Hamamatsu ORCA-ER C4742-95. *In vivo* time lapses were a Z-stack taken every 2–4 minutes for 30 min to 1.5 h. Image analysis was conducted with Freeware software ImageJ v1.46r, The ImageJ Plug-in ‘Grouped ZProjector’ was used to make projections of each frame.

## Supplementary Material

Supplementary Material
