# Supplementary material for: Activation of Drosophila hemocyte motility by the ecdysone hormone
Source: Biol Open. 2013 Nov 14;2(12):1412–20. doi: 10.1242/bio.20136619 (PMC3863427; doi:10.1242/bio.20136619)
Supplement: Supplementary Material [file supp_2_12_1412__index.html]

Activation of Drosophila hemocyte motility by the ecdysone hormone — Activation of Drosophila hemocyte motility by the ecdysone hormone — Supplementary Material 

# Activation of *Drosophila* hemocyte motility by the ecdysone hormone

## bio.20136619 Supplementary Material

**Files in this Data Supplement:**

- Supplementary Material - Christopher J. Sampson et al. doi: 10.1242/bio.20136619
- Movie 1 - **Movie 1. Time lapse of Late LIII hemocytes *in vivo*.** A late LIII w; pxn\_GAL4-UAS\_GFP; crq\_GAL4-UAS\_GFP larva was mounted on a glass slide using double-sided tape. The time lapse, of GFP-labelled hemocytes, was made by taking a Z-stack, with a slice interval of 4–6 µm, every 3 minutes for 1.5 hours. The video shows that at this stage, hemocytes are predominantly attached to the integument and there are very few hemocytes in circulation.
- Movie 2 - **Movie 2. Early stage LIII *Or-R* hemocytes in *ex vivo* culture on collagen IV matrix.** This live time-lapse is of a group of early stage LIII Or-R hemocytes that have not experienced a pulse of ecdysone *in vivo* or *ex vivo* conditions. The time lapse, of hemocytes in bright field, was constructed from live cell conditions with a frame rate of 1 frame/15 seconds taken over a minimum of a 20 minute time period.
- Movie 3 - **Movie 3. WPP *Or-R* hemocytes *ex vivo* on collagen IV matrix.** This live time-lapse is of a group of WPP Or-R hemocytes that have experienced an ecdysone pulse *in vivo* at the onset of metamorphosis. Live data collection was conducted *ex vivo* after hemocyte isolation. This time-lapse, of hemocytes in bright field, was constructed in the same way as in supplementary material Movie 2.
- Movie 4 - **Movie 4. Time lapse of WPP haemocytes *in vivo*.** An early w; pxn\_GAL4-UAS\_GFP; crq\_GAL4-UAS\_GFP WPP (1 h APF) was mounted on a glass slide using double-sided tape. The time lapse, of GFP-labelled hemocytes, was made by taking a Z-stack, with a slice interval of 4–6 µm, every 3 minutes for a minimum of 30 minutes. The video shows that at this stage, hemocytes are leaving the dorsal patches at the integument and migrating towards target tissues.
- Movie 5 - **Movie 5. Early stage LIII *Or-R* hemocytes isolated and incubated *ex vivo* with ecdysone hormone.** Early stage LIII hemocytes were incubated for 3.5 hours with 10% (v/v) ecdysone. This live time-lapse, of hemocytes in brightfield, was conducted, after the incubation period, for a minimum of 2 hours at a frame rate of 1 frame/15 seconds.
- Movie 6 - **Movie 6. Control *UAS-EcRB2(F645A)* only LIII hemocytes incubated *ex vivo* with ecdysone hormone.** Best representative early stage LIII hemocytes from UAS-EcR(F645A) only controls were isolated and incubated with 10% (v/v) ecdysone *ex vivo*. Hemocytes were incubated for 3.5 hours and then live time-lapse, of these hemocytes in brightfield, was conducted after incubation period for a minimum of 20 minutes at a frame rate of 1 frame/15 seconds.
- Movie 7 - **Movie 7. *He\_GAL4* driven *UAS-EcRB2(F645A)* LIII hemocytes isolated and incubated with ecdysone *ex vivo*.** Best representative early stage LIII hemocytes from *He\_GAL4* expressed *UAS-EcRB2(F645A)* time-lapsed after 3.5 hours ecdysone incubation *ex vivo*. This live time-lapse, of hemocytes in brightfield, was conducted after incubation period for a minimum of 20 minutes at a frame rate of 1 frame/15 seconds.
- Movie 8 - **Movie 8. Pxn\_GAL4/Crq\_GAL4 driven *UAS-EcRB2(F645A)* WPP hemocytes *in vivo*.** Hemocytes are highlighted with GFP driven by other copies of the GAL4 present in the w; pxn\_GAL4-UAS\_GFP; crq\_GAL4-UAS\_GFP line. The pupa was mounted on a glass slide using double-sided tape. The time lapse, of GFP-labelled hemocytes, was made by taking a Z-stack, with a slice interval of 4–6 µm, every 3 minutes for a minimum of 30 minutes, represented at 120× greater display speed. This time lapse shows that by expressing the dominant negative form of EcRB2, at the 1–1.5 h APF stage, there is an accumulation of hemocytes in the dorsal patches similar to that of a wild type Late LIII larva and a complete lack of migration when compared to a wild type early WPP.
- Movie 9 - **Movie 9. *HmL\_GAL4* driven *UAS-EcRB2(F645A)* LIII hemocytes isolated and incubated with ecdysone *ex vivo*.** Best representative early stage LIII hemocytes from *HmL\_GAL4* expressed *UAS-EcRB2(F645A)* time-lapsed after 3.5 hours ecdysone incubation *ex vivo*. This live time-lapse, of hemocytes in brightfield, was conducted after incubation period for a minimum of 20 minutes at a frame rate of 1 frame/15 seconds.
- Movie 10 - **Movie 10. *HmL\_GAL4* driven *UAS-EcRA(W650A)* LIII hemocytes isolated and incubated with ecdysone *ex vivo*.** Best representative early stage LIII hemocytes from *HmL\_GAL4* expressed *UAS-EcRA(W650A)* time-lapsed after 3.5 hours ecdysone incubation *ex vivo*. This live time-lapse, of hemocytes in brightfield, was conducted after incubation period for a minimum of 20 minutes at a frame rate of 1 frame/15 seconds.
