# Supplementary material for: Activation of Drosophila hemocyte motility by the ecdysone hormone
Source: Biol Open. 2013 Nov 14;2(12):1412–20. doi: 10.1242/bio.20136619 (PMC3863427; doi:10.1242/bio.20136619)
Supplement: Supplementary Material [file supp_bio.20136619_bio.20136619-s1.pdf]

Supplementary Material

Christopher J. Sampson et al. doi: 10.1242/bio.20136619

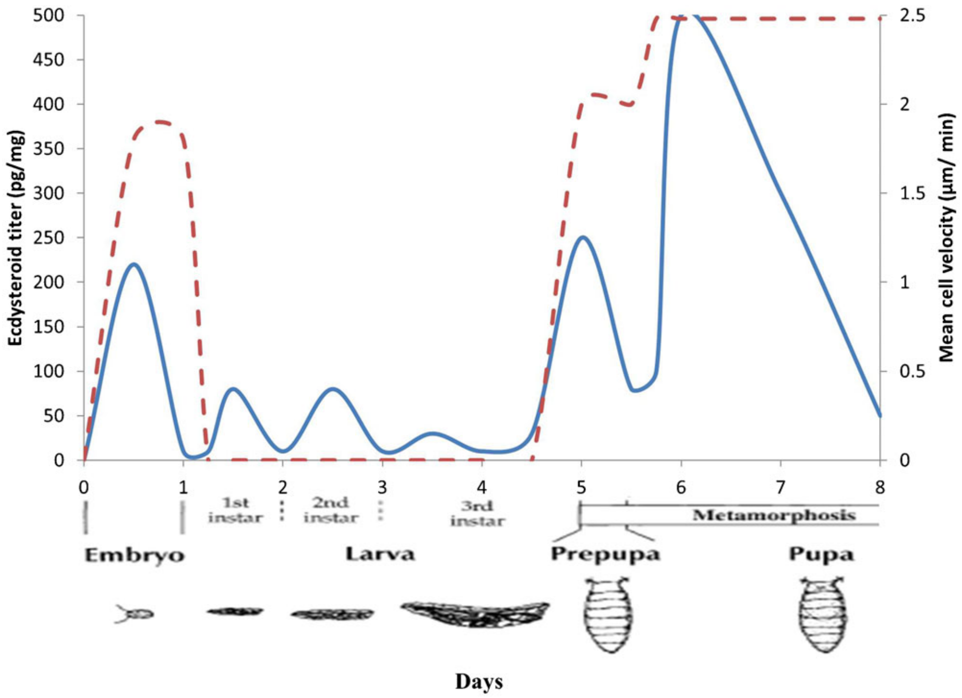

Fig. S1. Schematic representation of developmental ecdysone pulses and known hemocyte activity. Schematic representation of the ecdysone pulses that occur during the development of *Drosophila melanogaster* from embryonic to pupal life stages adapted from Thummel, 2001. The blue line shows the amounts of ecdysteroid (left Y axis) going through a series of peaks (pulses), following a temporal pattern. Red line, right Y axis, shows the mean cell migration velocity of hemocytes (μm/min) (Wood et al., 2006; Moreira et al., 2011; Sampson and Williams, 2012b) as a measure of hemocyte activity and motility. High activity and motility coincide with higher titres of ecdysteroid at embryogenesis and at the onset of metamorphosis.

Biology Open

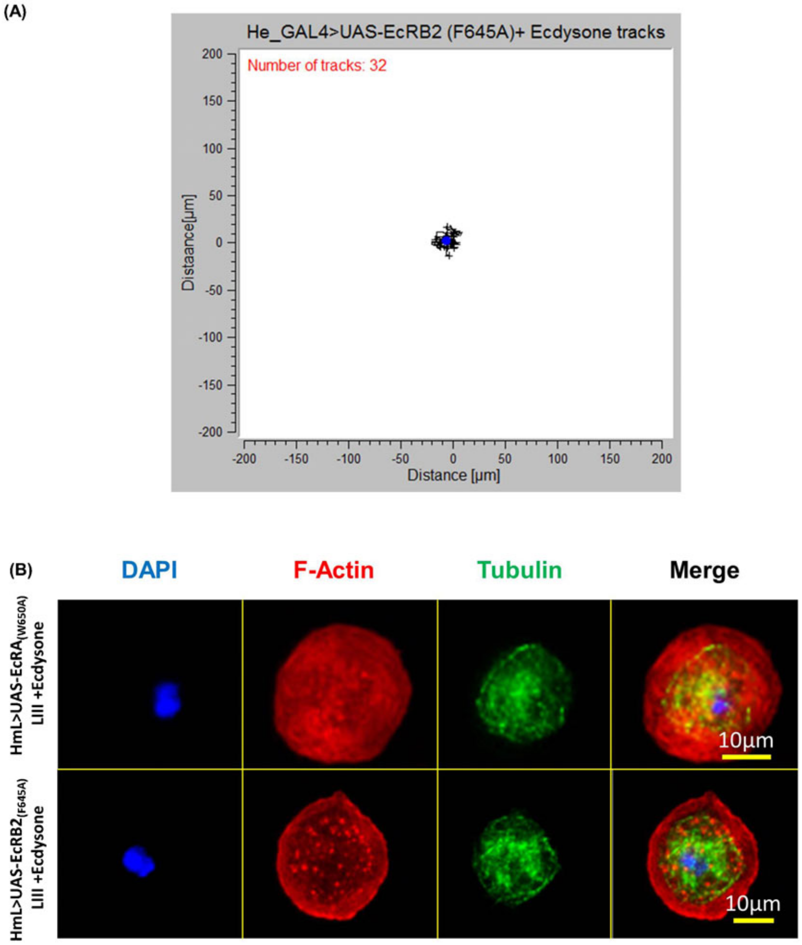

Fig. S2. Dominant negative forms of EcR deliver the same result, using either *He_GAL4* or *HmL_GAL4*. (A) Fixed point track plot of *He_GAL4* driven *UAS-EcRB2*$_{(F645A)}$ LIII + ecdysone hemocytes. Both X and Y axis refer to distance in µm and N refers to the number of individual cells tracked. Little or no movement is observed, similar to results obtained with *Hml_GAL4 UAS-EcRB2*$_{(F645A)}$ (compare with Fig. 4A). (B) Fixed hemocyte images, taken at 63× magnification, showing DAPI, F-actin, and β-tubulin staining. DAPI (blue) labels the nucleus of the cell, phalloidin (red) labels polymerised actin (F-actin), and β-tubulin (green) labels the microtubules of the cell; merged images on the right. The sampled hemocytes from *HmL_GAL4 UAS-EcR*$_{(W650A)}$ and *UAS-EcRB2*$_{(F645A)}$ look similar to *He_GAL4 UAS-EcRB2*$_{(F645A)}$ shown in Fig. 3.

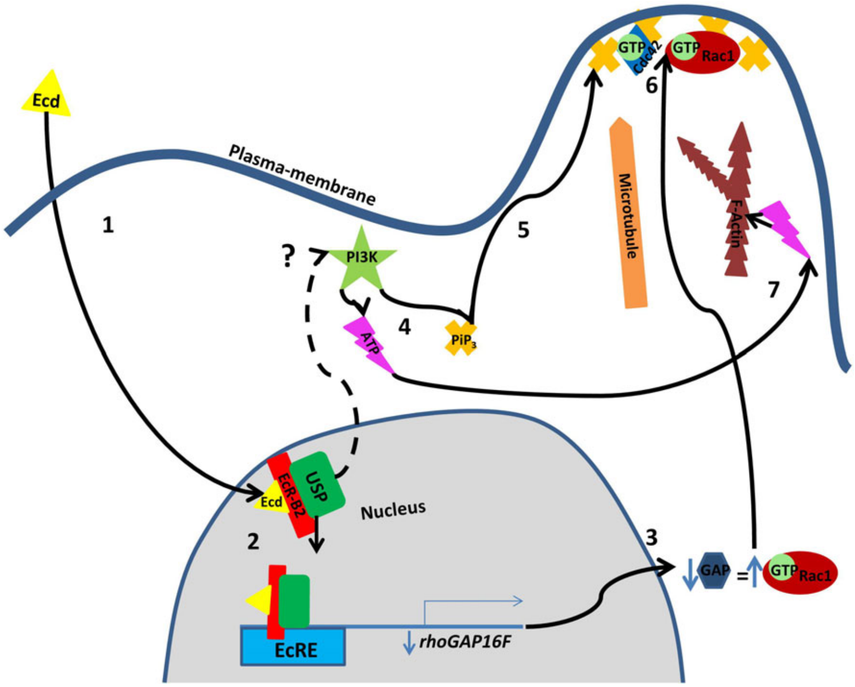

Fig. S3. Possible model of the molecular mechanism employed by ecdysone to induce cell activity. This figure represents a schematic model of various intra-cellular signalling events that leads to the activation of hemocytes, and increased motile behaviour, as derived from our results and published work regarding ecdysone. From the point that ecdysone is present in the media, the following steps occur: (1) Extra-cellular ecdysone (Ecd) diffuses and binds to the EcR-B2/USP receptor complex in the cell nucleus. (2) The receptor-ligand complex now binds to respective EcRE promoters to decrease the expression of *rhoGAP16F*. (3) By a decrease in *rhoGAP16F* expression, less Rac1 GAP is produced leading to a reduction in the overall concentration of this Rac1 GAP therefore shifting the Rho-GTPase switch toward more Rac1-GTP active molecules. (4) Ecdysone affects PI3K, whether directly or indirectly, symbolised by the dashed line, to increase levels of ATP and PiP_3 by autophagy. (5) PiP_3 now relocates to the plasma-membrane edge to allow for (6), the localisation of GTP-bound GTPases – Rac1 and Cdc42. The presence of GTP-Rac1 and GTP-Cdc42 induces signalling cascades that ultimately lead to (7), the polymerisation and extension of actin and tubulin, respectively, utilising the excessive pool of ATP, for actin polymerisation, produced from step 4.

Biology Open

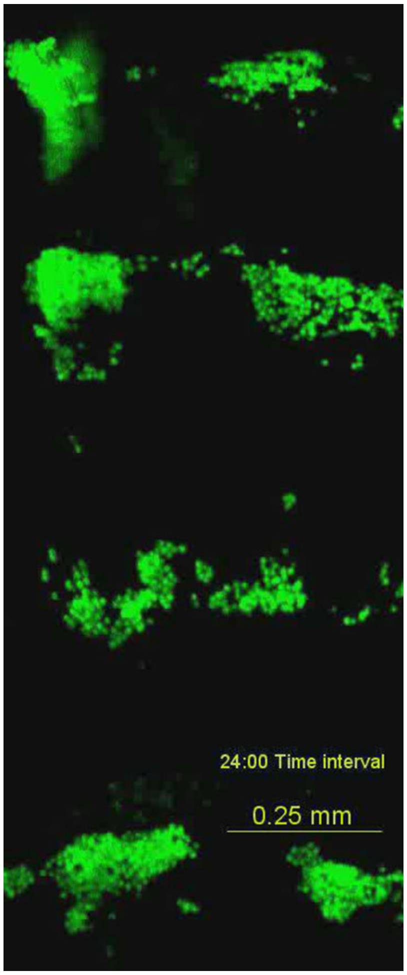

Movie 1. Time lapse of Late LIII hemocytes *in vivo*. A late LIII w; pxn_GAL4-UAS_GFP; crq_GAL4-UAS_GFP larva was mounted on a glass slide using double-sided tape. The time lapse, of GFP-labelled hemocytes, was made by taking a Z-stack, with a slice interval of 4–6 µm, every 3 minutes for 1.5 hours. The video shows that at this stage, hemocytes are predominantly attached to the integument and there are very few hemocytes in circulation.

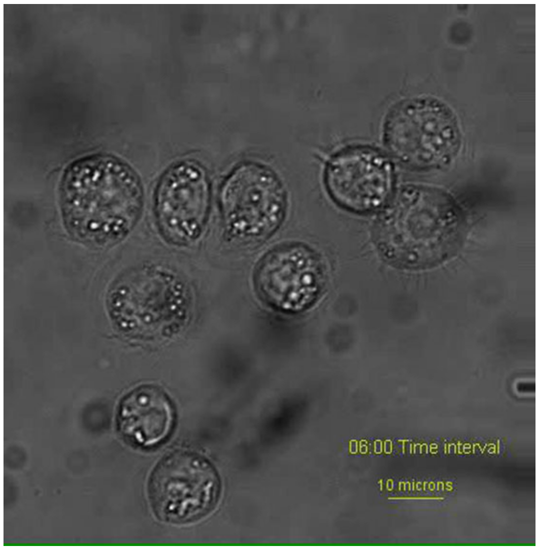

Movie 2. Early stage LIII *Or-R* hemocytes in *ex vivo* culture on collagen IV matrix. This live time-lapse is of a group of early stage LIII Or-R hemocytes that have not experienced a pulse of ecdysone *in vivo* or *ex vivo* conditions. The time lapse, of hemocytes in bright field, was constructed from live cell conditions with a frame rate of 1 frame/15 seconds taken over a minimum of a 20 minute time period.

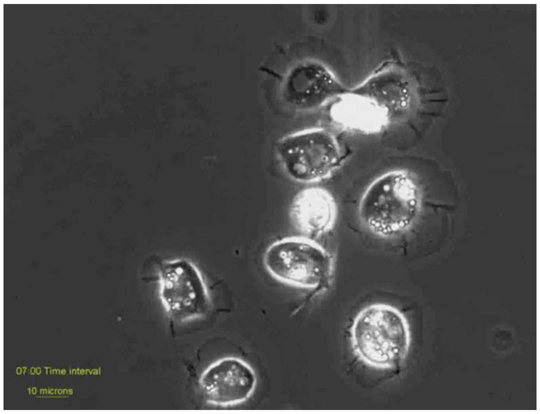

Movie 3. WPP *Or-R* hemocytes *ex vivo* on collagen IV matrix. This live time-lapse is of a group of WPP Or-R hemocytes that have experienced an ecdysone pulse *in vivo* at the onset of metamorphosis. Live data collection was conducted *ex vivo* after hemocyte isolation. This time-lapse, of hemocytes in bright field, was constructed in the same way as in supplementary material Movie 2.

Biology Open

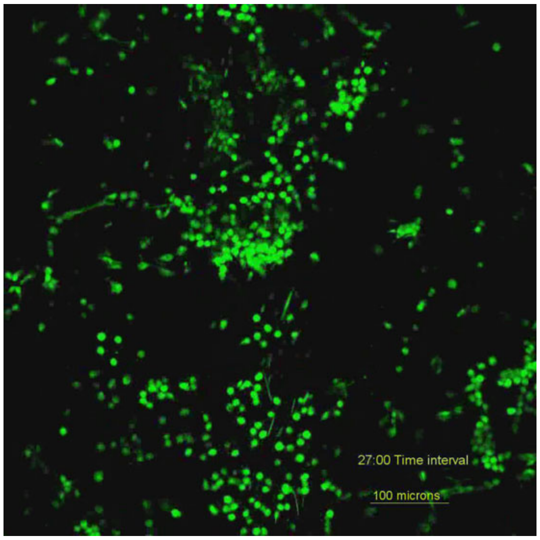

Movie 4. Time lapse of WPP haemocytes *in vivo*. An early w; pxn_GAL4-UAS_GFP; crq_GAL4-UAS_GFP WPP (1 h APF) was mounted on a glass slide using double-sided tape. The time lapse, of GFP-labelled hemocytes, was made by taking a Z-stack, with a slice interval of 4–6 µm, every 3 minutes for a minimum of 30 minutes. The video shows that at this stage, hemocytes are leaving the dorsal patches at the integument and migrating towards target tissues.

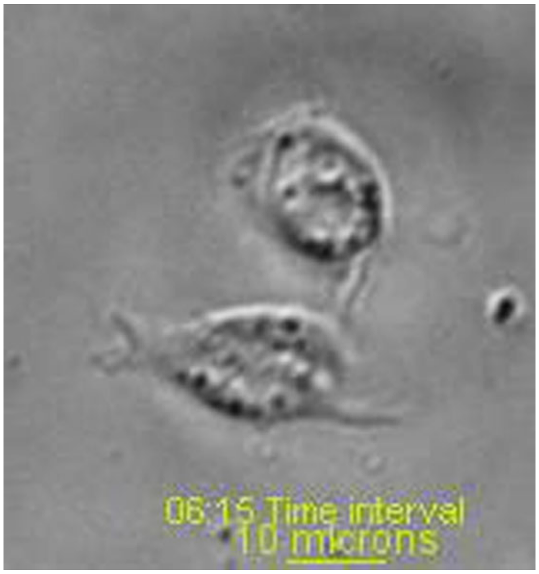

Movie 6. Control *UAS-EcRB2$_{(F645A)}$* only LIII hemocytes incubated *ex vivo* with ecdysone hormone. Best representative early stage LIII hemocytes from UAS-EcR$_{(F645A)}$ only controls were isolated and incubated with 10% (v/v) ecdysone *ex vivo*. Hemocytes were incubated for 3.5 hours and then live time-lapse, of these hemocytes in brightfield, was conducted after incubation period for a minimum of 20 minutes at a frame rate of 1 frame/15 seconds.

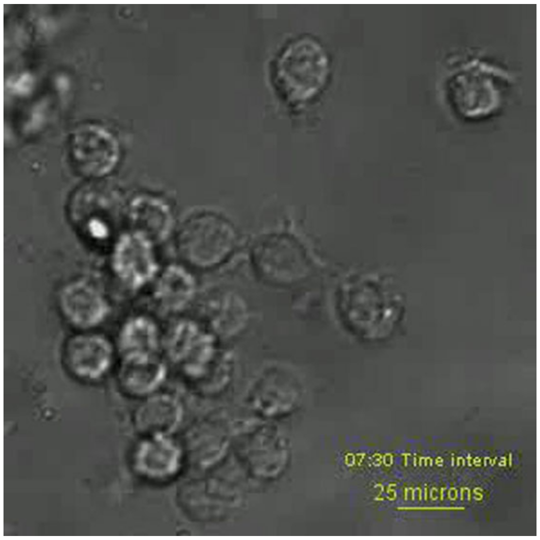

Movie 5. Early stage LIII *Or-R* hemocytes isolated and incubated *ex vivo* with ecdysone hormone. Early stage LIII hemocytes were incubated for 3.5 hours with 10% (v/v) ecdysone. This live time-lapse, of hemocytes in brightfield, was conducted, after the incubation period, for a minimum of 2 hours at a frame rate of 1 frame/15 seconds.

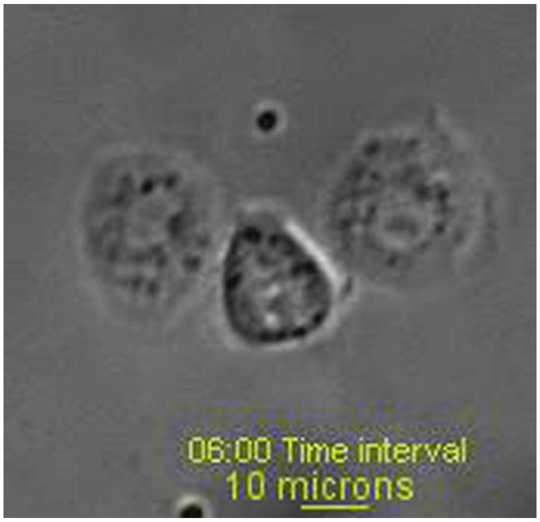

Movie 7. *He_GAL4* driven *UAS-EcRB2$_{(F645A)}$* LIII hemocytes isolated and incubated with ecdysone *ex vivo*. Best representative early stage LIII hemocytes from *He_GAL4* expressed *UAS-EcRB2$_{(F645A)}$* time-lapsed after 3.5 hours ecdysone incubation *ex vivo*. This live time-lapse, of hemocytes in brightfield, was conducted after incubation period for a minimum of 20 minutes at a frame rate of 1 frame/15 seconds.

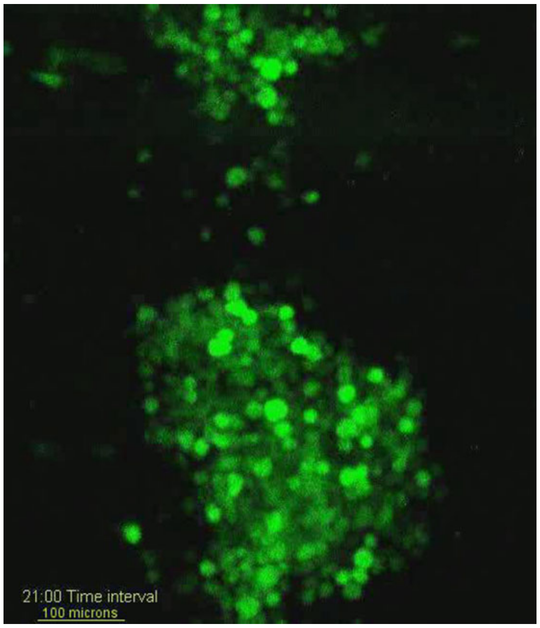

Movie 8. Pxn_GAL4/Crq_GAL4 driven *UAS-EcRB2$_{(F645A)}$* WPP hemocytes *in vivo*. Hemocytes are highlighted with GFP driven by other copies of the GAL4 present in the w; pxn_GAL4-UAS_GFP; crq_GAL4-UAS_GFP line. The pupa was mounted on a glass slide using double-sided tape. The time lapse, of GFP-labelled hemocytes, was made by taking a Z-stack, with a slice interval of 4–6 µm, every 3 minutes for a minimum of 30 minutes, represented at 120× greater display speed. This time lapse shows that by expressing the dominant negative form of EcRB2, at the 1–1.5 h APF stage, there is an accumulation of hemocytes in the dorsal patches similar to that of a wild type Late LIII larva and a complete lack of migration when compared to a wild type early WPP.

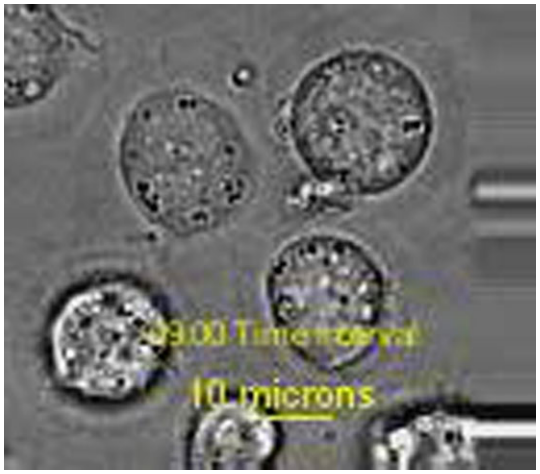

Movie 9. *HmL_GAL4* driven *UAS-EcRB2$_{(F645A)}$* LIII hemocytes isolated and incubated with ecdysone *ex vivo*. Best representative early stage LIII hemocytes from *HmL_GAL4* expressed *UAS-EcRB2$_{(F645A)}$* time-lapsed after 3.5 hours ecdysone incubation *ex vivo*. This live time-lapse, of hemocytes in brightfield, was conducted after incubation period for a minimum of 20 minutes at a frame rate of 1 frame/15 seconds.

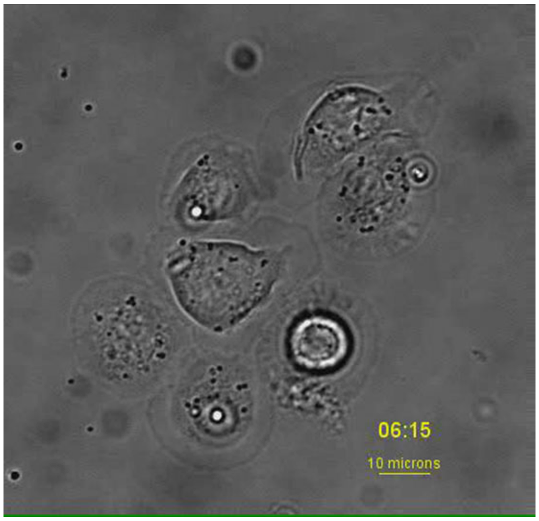

Movie 10. *HmL_GAL4* driven *UAS-EcRA$_{(W650A)}$* LIII hemocytes isolated and incubated with ecdysone *ex vivo*. Best representative early stage LIII hemocytes from *HmL_GAL4* expressed *UAS-EcRA$_{(W650A)}$* time-lapsed after 3.5 hours ecdysone incubation *ex vivo*. This live time-lapse, of hemocytes in brightfield, was conducted after incubation period for a minimum of 20 minutes at a frame rate of 1 frame/15 seconds.

Biology Open
